# Data of *de novo* assembly and annotation of transcriptome from *Aspidistra fenghuangensis* (Asparagaceae: Nolinoideae)

**DOI:** 10.1016/j.dib.2020.105738

**Published:** 2020-05-21

**Authors:** Ran Meng, Ji-Yuan Zhang, Dai-Gui Zhang, Ze-Long Nie, Ying Meng

**Affiliations:** Key Laboratory of Plant Resources Conservation and Utilization, College of Biological Resources and Environmental Sciences, Jishou University, Jishou, 416000, China

**Keywords:** Aspidistra fenghuangensis, Transcriptome analysis, De novo assembly, Functional annotation

## Abstract

*Aspidistra* is a large genus of herbaceous plants with more than 130 species growing in tropical forests of SE Asia and specially diversified in southern China and northern Vietnam. The genus is characterized by its evergreen understorey habitats with flowers set at ground level and more or less hidden in litter material. *Aspidistra fenghuangensis* is a species currently only known from central China. In recent years, number of species in this genus has been greatly increased. However, the high throughput sequencing data have never been reported in this genus. Here, we sequenced the transcriptome of *A. fenghuangensis* obtained from young leaves using the Illumina HiSeq2000 with 9.15Gb of clean data. Because of the absence of a reference-grade genome in the genus, a *de novo* assembly of the transcriptome data with full annotation have been carried out. This data is accessible via NCBI BioProject (PRJNA608213).

Specifications Table

**Table:** 

Subject	Plant Science
Specific subject area	Transcriptomics
Type of data	Table, figure
How data were acquired	Illumina HiSeq 2000
Data format	Raw, analyzed
Parameters for data collection	Total RNA of *Aspidistra fenghuangensis* was extracted from young leaves and used for cDNA library construction. Paired-end reads were generated using Illumina HiSeq 2000 system. Functional annotation using BLAST searches and BLAST2GO against databases including NCBI non-Redundant (nr) protein database, SwissProt, Pfam, KOG/COG/eggNOG, KEGG, GO.
Description of data collection	After pre-processing of the clean reads (9.15Gb, 30,779,512 reads) and *de novo* assembling of dataset, a total of 149,288 transcripts and 57,501 unigene were found, 27173 unigenes were annotated.
Data source location	Institution: Jishou University City/Town/Region: Gaowangjie Nature Reserve in Guzhang, Hunan Country: China Latitude and longitude (and GPS coordinates) for collected samples/data: 28°43′6.96" N, 110°2′54.24" E
Data accessibility	Repository name: NCBI (National Center for Biotechnology Information) Data identification number: SRR11190988 Direct URL to raw data of sequence: https://trace.ncbi.nlm.nih.gov/Traces/sra/?run=SRR11190988 Direct URL to data of functional annotation: https://data.mendeley.com/datasets/sgw32nkhyd/2

## Value of the Data

• To our knowledge, this is the first *de novo* transcriptome that significantly increased amount of genomic information available for this plant, also useful as reference to other *Aspidistra* species.
• This data will be helpful to perform phylogenomic studies of *Aspidistra* species as well as backbone relationships within Nolinoideae, a complex subfamily of Asparagaceae.
• Further, this assembled data and functional annotation will serve as a reference for future studies including the research fields of identification of metabolic pathways and gene expression in *Aspidistra* and other related species in this genus.

## 1 Data

**Fig. 1:**
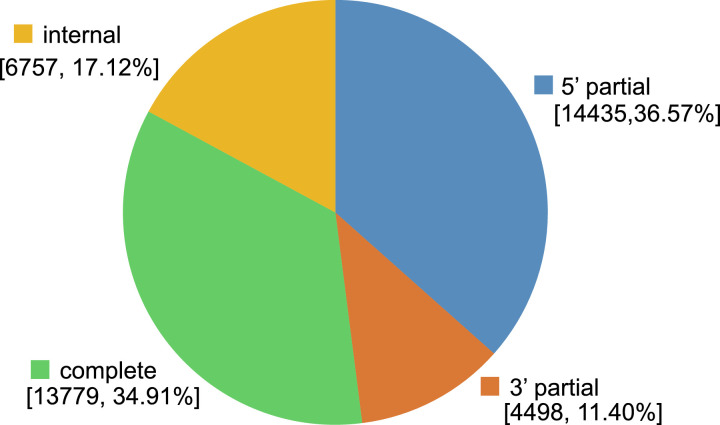
Types of coding sequences predicted from the unigenes of *A. fenghuangensis*. Complete = presence of start and stop codons in the open reading frames (ORF); 5’ partial = missing stop codon the ORF; 3’ partial = missing start codon in the ORF; Internal = neither start nor stop codons were detected in the ORF.

**Fig. 2:**
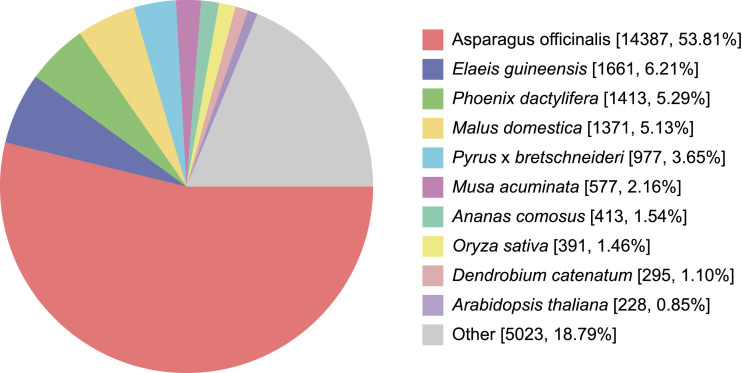
Distribution of species for best BLAST hits of the *A. fenghuangensis* transcripts against the NCBI nr database with a cut off E-value 1e^−5^.

The transcriptomic data of *Aspidistra fenghuangensis* was sequenced for the first time using Illumina Hiseq 2000. The sequencing run generated a total of 9.15 GB (30,779,512 reads) clean data in FASTQ format, which has been deposited in the SRA database (PRJNA608213). A *de novo* assemble of the clean reads was performed with relative information summarized in Table 1. The analysis showed that a total of 39,469 unigenes contained putative open reading frames (ORFs) and coding sequences (CDS). Among them, 34.91 % of CDS had a complete ORF which containing defined start and stop codons (Fig. 1). Other than that, 25,690 transcripts were classified as partial CDS. Specifically, 14,435 transcripts were classified as “5’ partial” containing a stop codon and missing start codon, 4,498 were grouped as “3’ partial” containing a start codon and lacking stop codon, and 6,757 were categorised as “internal” with missing of both the start and stop codons. Among the 57501 unigenes, a total of 27,173 was annotated using multiple methods and databases with a statistic overview of the annotation in Table 2 and details presented in Supplementary material S1. For the species distribution of the 26,758 unigenes annotated against NCBI nr database, 53.81% are from *Asparagus officinalis* of Asparagaceae (Fig. 2).

**Table 1:** Transcripts and Unigene statistics for the leaf transcriptome of *Aspidistra fenghuangensis*.

	Transcript	Unigene
Total Number	149288	57501
Total Length	165068535	47020461
N50 Length	1563	1131
Mean Length	1105.705315	817.7329264

**Table 2:** Unigene annotated statistics of *Aspidistra fenghuangensis.*

	Annotated number	300 <= length < 1000	Length >= 1000
COG	7801	3130	4671
GO	14909	8332	6577
KEGG	10148	5426	4722
KOG	15228	8112	7116
Pfam	17587	8251	9336
Swissprot	18027	9548	8479
eggNOG	24414	13528	10886
NCBI nr	26758	15523	11235
All annotated	27173	15893	11280

## 2 Experimental design, materials, and methods

### 2.1 Sample collection

Healthy and approximately 3-year-old plants of *Aspidistra fenghuangensis* were collected from Gaowangjie Nature Reserve in Guzhang, Hunan, China. To reduce sampling variation, young leaves were pooled from three plants and immediately submerged into liquid nitrogen for transportation.

### 2.2 cDNA library construction and sequencing

A total amount of 1 μg RNA per sample was used as input material for the RNA sample preparations. Sequencing libraries were generated using NEBNext®Ultra™ RNA Library Prep Kit for Illumina®(NEB, USA) following manufacturer's recommendations and index codes were added to attribute sequences to sample. At last, PCR products were purified (AMPure XP system) and library quality was assessed on the Agilent Bioanalyzer 2100 system.

The clustering of the index-coded samples was performed on a cBot Cluster Generation System using TruSeq PE Cluster Kit v3-cBot-HS (Illumia) according to the manufacturer's instructions. After cluster generation, the library preparations were sequenced on an Illumina Hiseq 2000 platform and paired-end reads were generated.

### 2.3 Sequence data assembly and bioinformatics analysis

Raw data with fastq format were firstly filtered and cleaned with Fastp [1]. Clean reads were obtained by removing reads containing adapter, reads containing ploy-N and low quality reads from raw data. All the downstream analyses were based on clean data with high quality. Transcriptome assembly was accomplished based on the Aspidistra_fenghuangensis_1.fq and Aspidistra_fenghuangensis_2.fq using Trinity [2] with min_kmer_cov set to 2 and all other default parameters. Transcripts were then subjected for clustering using CD-HIT-EST with an identity more than 90% and coverage of 100% [3]. We further determined the completeness of our unigene dataset with Bench-marking universal single-copy orthologs (BUSCO) software version 4 with the database of liliopsida_odb10 [4].

BLAST searches were performed based on similarity to known protein sequences in NCBI nr database to detect whether the dataset is subject to excessive exogenous contamination, with an adjusted E-value = 1e^−5^ [5]. In addition, both Swiss-Prot (A manually annotated and reviewed protein sequence database) [6] and Pfam (Protein family) databases are used to categorise the transcripts. Other gene function annotations include GO (Gene Ontology) [7], KEGG (Kyoto Encyclopedia of Genes and Genomes) [8] and KOG/COG/eggNOG (Clusters of Orthologous Groups of proteins) [9,10] were conducted using BLAST2GO with default parameters. TransDecoder [11] was used to recognize ORFs within CDS of at least 100 amino acids in length from the assembled transcripts with comparison of Pfam database.

## Declaration of Competing Interest

The authors declare that they have no known competing financial interests or personal relationships which have, or could be perceived to have, influenced the work reported in this article.

## Appendix Supplementary materials

Image, application 1
